# Qualifying for Practice

**Published:** 1896-04-18

**Authors:** 


### QUALIFYING FOR PRACTICE.

We have been asked the question whether the London M.B. degree absolutely qualifies a man to practice in England without any other qualification whatsoever. Certainly it does. Any diploma or degree which admits to the Register qualifies for practice in any part of the United Kingdom. It was in consequence of the fact that no partial qualification would admit to the Register that the conjoint diplomas were introduced, but the universities were able to examine in all the subjects. Some, such as the University of London, included surgery in the examination for the M.B., thus making it a complete qualification; others, such as Aberdeen, Edinburgh, and Glasgow, attained the same end by refusing to grant either the M.B. or Ch.B. separately. The same end is attained but by different means, the only balance in favour of the Scotchmen being that they have more letters to their name.

